# Human protein Staufen-2 promotes HIV-1 proliferation by positively regulating RNA export activity of viral protein Rev

**DOI:** 10.1186/1742-4690-11-18

**Published:** 2014-02-13

**Authors:** Atoshi Banerjee, Ronald Benjamin, Kannan Balakrishnan, Payel Ghosh, Sharmistha Banerjee

**Affiliations:** 1 Department of Biochemistry, School of Life Sciences, University of Hyderabad, Gachibowli, Hyderabad, Andhra Pradesh 500046, India; 2 Department of Biotechnology, University of Pune, Ganeshkhind, Pune, Maharashtra 411007, India

**Keywords:** Staufen-2, Rev, RRE transport, HIV-1

## Abstract

**Background:**

The export of intron containing viral RNAs from the nucleus to the cytoplasm is an essential step in the life cycle of Human Immunodeficiency Virus-1 (HIV-1). As the eukaryotic system does not permit the transport of intron containing RNA out of the nucleus, HIV-1 makes a regulatory protein, Rev, that mediates the transportation of unspliced and partially spliced viral mRNA from the nucleus to the cytoplasm, thereby playing a decisive role in the generation of new infectious virus particles. Therefore, the host factors modulating the RNA export activity of Rev can be major determinants of virus production in an infected cell.

**Results:**

In this study, human Staufen-2 (hStau-2) was identified as a host factor interacting with HIV-1 Rev through affinity chromatography followed by MALDI analyses. Our experiments involving transient expressions, siRNA mediated knockdowns and infection assays conclusively established that hStau-2 is a positive regulator of HIV-1 pathogenesis. We demonstrated that Rev-hStau-2 interactions positively regulated the RNA export activity of Rev and promoted progeny virus synthesis. The Rev-hStau-2 interaction was independent of RNA despite both being RNA binding proteins. hStau-2 mutant, with mutations at Q314R-A318F-K319E, deficient of binding Rev, failed to promote hStau-2 dependent Rev activity and viral production, validating the essentiality of this protein-protein interaction. The expression of this positive regulator was elevated upon HIV-1 infection in both human T-lymphocyte and astrocyte cell lines.

**Conclusions:**

With this study, we establish that human Staufen-2, a host factor which is up-regulated upon HIV-1 infection, interacts with HIV-1 Rev, thereby promoting its RNA export activity and progeny virus formation. Altogether, our study provides new insights into the emerging role of the Staufen family of mRNA transporters in host-pathogen interaction and supports the notion that obliterating interactions between viral and host proteins that positively regulate HIV-1 proliferation can significantly contribute to anti-retroviral treatments.

## Background

Human Immunodeficiency Virus-1 (HIV-1) is a pathogenic retrovirus that cripples the human immune system making it susceptible to several opportunistic infections and malignancies, leading to Acquired Immunodeficiency Syndrome (AIDS) [1-4]. Like all other successful intracellular pathogens, HIV also establishes dynamic biological interactions with the host cell machinery for persistence and pathogenesis. HIV-1, during the course of infection, makes completely spliced, partially spliced and unspliced mRNAs for the synthesis of viral proteins to generate new virion particles [5]. While completely spliced viral mRNA utilizes the constitutive mRNA nuclear export pathway of the eukaryotic cells [6], the partially spliced and the unspliced viral mRNAs are retained in the nuclei of the infected host cells for further splicing or degradation. HIV overcomes this problem by synthesizing a protein called Rev (Regulator of virion expression) from completely spliced viral mRNA [7]. Rev is a 18 kDa nucleo-cytoplasmic transport protein that tethers to Rev responsive element (RRE) present within the partially spliced and the unspliced viral mRNA and facilitate their export to the cytoplasm for the translation and progeny virus synthesis [8]. Rev, therefore, is an essential protein of the virus, the inactivation or deficiency of which results in failure to produce new virion particles [5].

Under physiological conditions, Rev shuttles between the cytoplasmic and the nuclear compartments where it interacts with several host factors that modulate its activity, consequently affecting HIV proliferation. Host nuclear proteins such as Matrin-3, RNA helicases, PSF, Rab/hRIP and CRM-1/exportin-1 that interact with Rev mediate its export from the nucleus to the cytoplasm [9-16]. Host proteins, such as, Ataxia-telangiectasia-mutated (ATM) protein [17], eukaryotic Initiation Factor-5a [18], Sam68 [19] and DEAD box RNA helicases [20], function as positive effectors of Rev activity, while factors such as, anti-apoptotic mitochondrial protein, HS1-associated protein X-1 (Hax-1), negatively regulate the function of HIV-1 Rev by perturbing its sub-cellular distribution [21]. Some host proteins, which prevent effective binding of Rev to RRE, such as Rev Response Element Binding Protein of 49 kDa (RREBP49) [22] or Alternate splicing factor (SF2/ASF) [23] interferes with the export activity of Rev, thereby reducing the virion particle formation. Apart from host factors, viral proteins also play a role in the sub-cellular localization of Rev which can eventually affect its activity [24]. An exhaustive list of the possible host factors interacting with HIV-1 has been reported by Jager et. al [25].

Acting as a post-transcriptional regulator of HIV-1 gene expression, identifying the cellular factors modulating Rev activity is highly pertinent to understand the pathogenicity of HIV-1. We hypothesized that cell specific host factors modulating the activity of Rev can serve as determinants in viral propagation and pathogenesis. Therefore, we attempted to capture Rev-interacting human cellular factors by making a Rev-affinity column, where purified recombinant histidine tagged HIV-1 Rev was used as bait. We characterized the interaction between Rev and a double-stranded RNA-binding host protein Staufen-2, identified from SUP-T1 (Human T cell lymphoblastoma) cell lysates. Human Staufen homolog 2 (hStau-2) is a double-stranded-RNA binding protein, with multiple splice variants [26] that possibly, like other mammalian Staufen homologs, forms ribonucleoprotein complexes (RNPs) [27]. hStau-2 has been shown to participate in targeted packaging and localization of mRNA in polarized cells and is a part of stress granules in neurons. hStau-2 has also been observed in other cell types including cell lines like HeLa and 293 F [28,29].

In this study, the intracellular interactions between Rev and hStau-2 were validated by transient expression of both the proteins in cell lines followed by co-immunoprecipitations. Ectopic and transient expressions, sub-cellular localizations, siRNA knockdowns and infection assays using wild type and mutant hStau-2 confirmed that the interaction of hStau-2 with Rev positively regulated the RRE containing RNA export activity of Rev and promoted progeny virus synthesis. Furthermore, HIV-1 infection elevated the expression of hStau-2 in both human T-lymphocyte and astrocyte cell lines. With these observations, we conclude that hStau-2, better known for its role in mRNA transportation and stress granule formation in neural cells [30], assumes a novel regulatory role during HIV-1 infection contributing to our existing knowledge of HIV-host interactions.

## Results

### Human Staufen-2 (hStau-2) interacted with HIV-1 Rev protein in RNA independent manner

**Figure 1:**
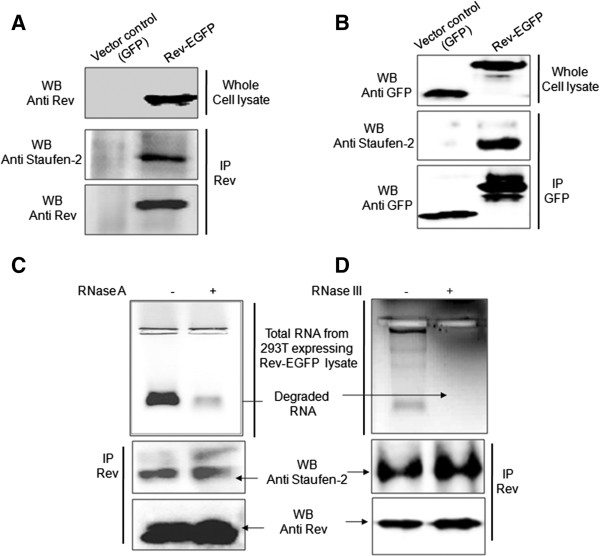
**Rev interacts with hStau-2. A)** Co-immunoprecipitation of endogenous hStau-2 with Rev-GFP using anti-Rev antibody and Western Blot with anti-hStau-2 antibody: HEK293T cells were transfected with Rev-EGFP vector or GFP vector control, cell lysates were prepared after 48 hours followed by IP and WB. Whole cell lysates were checked for Rev expression by anti-Rev antibody. **B)** Co-immunoprecipitation of endogenous hStau-2 with Rev-GFP using anti-GFP antibody and Western Blot with anti-hStau-2 antibody: HEK293T cells were transfected with Rev-EGFP vector or GFP vector control, cell lysates were prepared after 48 hours followed by IP and WB. Whole cell lysates were checked for GFP/Rev-GFP expression by anti-GFP antibody. **C)** RNase A treatment and pulldowns: Cell lysates were treated with RNase A. RNA degradation was assessed by RNA isolation from both RNase A untreated and treated cell lysates and run on 2% Agarose gel (1^st^ Panel). Treated and untreated samples were used for IP with anti-Rev antibody and WB by anti-Staufen-2 antibody (2^nd^ Panel) and anti-Rev antibody (3^rd^ Panel). **D)** RNase III treatment and pulldowns: Cell lysates were treated with RNase III. RNA degradation was assessed by RNA isolation from both RNase III untreated and treated cell lysates and run on 2% Agarose gel (1^st^ Panel). Treated and untreated samples were used for IP with anti-Rev antibody and WB by anti-Staufen-2 antibody (2^nd^ Panel) and anti-Rev antibody (3^rd^ Panel).

Purified recombinant polyhistidine tagged HIV-1 Rev (Additional file 1: Figure S1A), immobilized on Talon resin, was used to pull-down Rev interacting host factors from SUP-T1 (Human T cell lymphoblastoma) cell lysates (Additional file 1: Figure S1B). An endogenously expressed double stranded RNA binding host protein, human Staufen-2 (hStau-2), was identified after MALDI analyses as one of the proteins in pull-down samples showing significant MASCOT score (Additional file 1: Figure S1C and Additional file 2: Figure S2). The interaction was further confirmed by co-immunoprecipitation of endogenously expressed hStau-2 from HEK293T cell lysates with transiently expressed GFP-tagged Rev (Rev-GFP). Rev-GFP was immunoprecipitated with mouse anti-Rev antibody and hStau-2 was detected by Western blot in the immunoprecipitated samples using goat anti-hStau-2 antibody (Figure 1A). HEK293T cells transfected with empty GFP vector was used as a control and did not show any hStau-2 signal (Figure 1A). The same was confirmed by immunoprecipitation with anti-GFP antibody followed by Western Blot with hStau-2 antibody (Figure 1B). These data clearly demonstrated that transiently expressed Rev could interact with the endogenous hStau-2 intracellularly in HEK293T cells.

hStau-2 and Rev, both are RNA binding proteins. We, therefore, questioned if the cellular RNA mediated the Rev-hStau-2 interactions. For this, Rev-GFP and hStau-2 were transiently co-expressed in HEK293T cells and the cell lysates were divided into 2 aliquots, one of which was treated with RNase A. Total RNA from the treated and the untreated samples were isolated and fractionated on 2% agarose gel to check for the efficiency of RNase A treatment (Figure 1C 1^st^ panel). The treated and the untreated lysates were then used for immunoprecipitation with mouse anti-Rev antibody followed by Western blot with goat anti-hStau-2 antibody. It was observed that the degradation of ssRNA did not affect the interactions between Rev and hStau-2 (Figure 1C 2^nd^ and 3^rd^ panels). Since hStau-2 is a dsRNA binding protein and Rev is also reported to bind to the structured RRE element, we checked if the interaction is mediated by any dsRNA. For this, cell lysates were treated with RNase III which degrades double strand RNA (Figure 1D 1^st^ panel). Immunoprecipitations were performed as mentioned above and hStau-2 was detected in the immunoprecipitated samples (Figure 1D 2^nd^ and 3^rd^ panels). It was clear from this experiment that Rev and hStau-2 interaction is not mediated by dsRNA either. With this, we concluded that HIV-1 Rev interaction with hStau-2 is not RNA dependent.

### Rev influenced the sub-cellular localization of hStau-2

**Figure 2:**
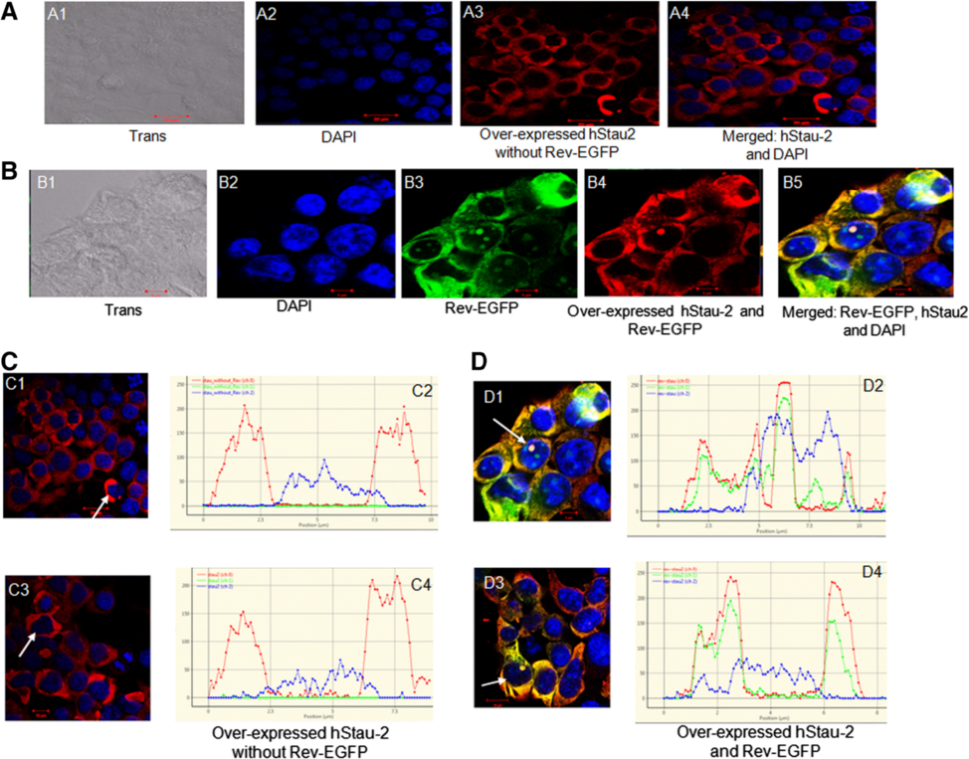
**Localization of Rev and Staufen-2 (hStau-2). A)** hStau-2 localizes in the cytoplasm in the absence of Rev: HEK293T cells were transfected with Staufen-2-59-CMV vector and stained with goat anti-Staufen-2, Rabbit anti-goat Alexa Fluor 568 antibody and nuclear staining by DAPI. **B)** hStau-2 co-localizes with Rev in both the nucleoli and the cytoplasm: HEK293T cells were transfected with Rev-EGFP and Staufen-2-59-CMV vector. Localization of GFP-tagged Rev was determined by green fluorescence, hStau-2 was determined by goat anti-Staufen-2, Rabbit anti-goat Alexa Fluor 568 antibody and nucleus was stained by DAPI. Merged images show co-localization of hStau-2 and Rev. **C)** Graphical representation of hStau-2 localization in the absence of Rev: Representative HEK293T cells overexpressing hStau-2 were (C1, C3) used for the distribution plots (C2, C4) using Huygens Essential software twin slicer tool, where blue line denotes the boundaries of the nucleus and region outside the blue line represent the cytoplasmic region. Red lines in the plots indicate hStau-2 localization. **D)** Graphical representation of hStau-2 localization in the presence of Rev: Representative HEK293T cells over-expressing hStau-2 and Rev-EGFP (D1, D3) were used for distribution plots (D2, D4) using Huygens Essential software twin slicer tool, where blue line denotes the boundaries of the nucleus and the region outside the blue line represent the cytoplasmic region. Green lines in the plots indicate Rev localization and Red lines indicate hStau-2 localization. The precise cells whose graphical analyses have been shown are indicated by the white arrows.

Rev mediates the transport of RRE-containing viral RNA across the nuclear membrane by shuttling between the nucleus and the cytoplasm of an infected cell [31]. hStau-2, though primarily localizes in the cytoplasm, has also been shown to shuttle between the nucleus and the cytoplasm [26,32]. With both the proteins exhibiting nucleo-cytoplasmic shuttling properties, we addressed the question if Rev and hStau-2 co-localize in these sub-cellular compartments. The cellular localization of hStau-2 alone and along with transiently expressed Rev-GFP was determined in HEK293T cells by confocal microscopy (Figure 2). When hStau-2 was overexpressed alone in HEK293T cells, it remained localized to the cytoplasm of the cells (Figure 2 A3-A4). However, when hStau-2 was co-expressed with Rev-GFP, a fraction of hStau-2 was also observed to be localized in the nucleoli along with Rev (Figure 2 B3-B5). The localization of the two proteins was graphically represented by using twin slicer tool of Huygens Essential software as discussed in the methodology section [24]. The nuclear margins are indicated by blue (DAPI staining), hStau-2 is represented by Red (Alexa Fluor 568) and Rev is shown by green (GFP). The plots clearly show that hStau-2 when expressed alone resides primarily in the cytoplasm while in the presence of Rev it is found in both the nucleoli and the cytoplasm (Figure 2C-D). The merged Rev-GFP and hStau-2 images (Figure 2 B5 and Figure 2D plots D2 and D4) show that the two proteins co-localize when expressed together. With this it was evident that the localization of hStau-2, which was primarily cytoplasmic, was influenced in the presence of Rev.

### HIV-1 infection up-regulated hStau-2 expression in human T-lymphocyte and astrocyte cell lines

**Figure 3:**
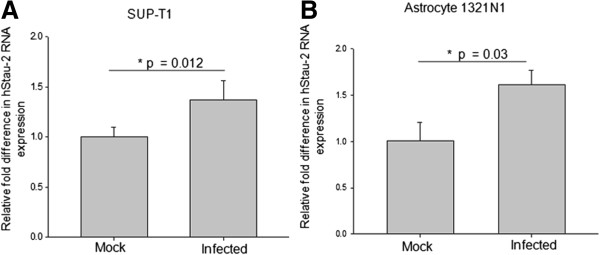
**hStau-2 expression in SUP-T1 and 1321 N1 cell lines upon HIV-1 infection.** Cells were infected with 50 ng/ml of p24 equivalent of NL4-3 virus. RNA was isolated from both mock and infected cells and qRT-PCR was carried out. **A)** hStau-2 expression in SUP-T1 cells **B)** hStau-2 expression in 1321 N1 cells. The result are from 4 independent experiments and error bars represent ± SD. *p value ≤ 0.05 was taken as significant. The data were normalized to the transcript levels of β–actin.

Human Staufen-1 has been implicated in encapsidation and assembly of HIV-1 [33-35] whereas both the mammalian homologs Staufen-1 and Staufen-2 are recruited in the stress granules of neural cells [30,36]. Apart from the neural cells, Staufen homologs have also been shown in other cell types [28,29]. Before any further characterization of Rev-hStau-2 interactions, we checked the fate of hStau-2 expression upon HIV-1 infection. Two HIV-1 permissive human cell-lines, SUP-T1 (Human T cell lymphoblastoma) and 1321 N1 (human astrocytoma) were used to verify the differential expression of hStau-2 upon HIV-1 (NL4-3) infection by quantitative Real time PCR. hStau-2 expression increased upon NL4-3 virus infection by 37 ± 18% in SUP-T1 (Figure 3A) while 60 ± 15% in astrocyte 1321 N1 (Figure 3B) when compared to the mock infection. The transcript levels of β-actin were used for normalization of the data.

### hStau-2 increased HIV-1 production

**Figure 4:**
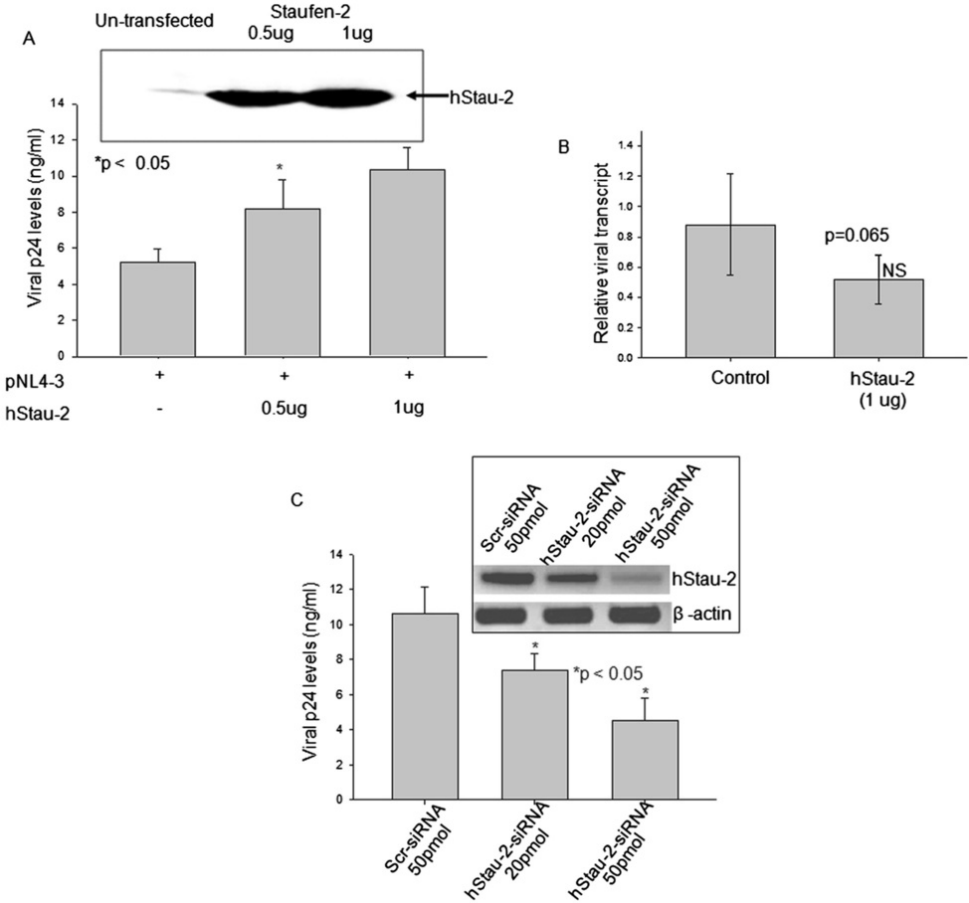
**Effect of hStau-2 expression on viral p24 levels. A)** Over-expression of hStau-2 increased HIV-1 production: hStau-2 over-expressed HEK293T cells or control cells were transfected with pNL4-3 pro-viral DNA and scored after 48 hours for p24 by ELISA in the culture supernatant. The inset shows over-expression of hStau-2 after transfection with increasing concentration of hStau2-59 construct. There was a significant increase in the p24 levels when 0.5 and 1ug of hStau2-59 construct was used. **B)** The relative quantification of full length (9 kb) viral transcript. Viral transcripts were quantified from hStau-2 overexpressed and control cells by qRT-PCR and normalized to β-actin. **C)** siRNA mediated knockdown of hStau-2 reduced HIV-1 production: hStau-2 siRNA or scrambled siRNA were transfected one day prior to pNL4-3 transfection in HEK293T cells. Inset: Semiquantitative RT-PCR gel showing a decrease in hStau-2 expression when hStau-2 specific siRNA was used. The viral p24 levels in the culture supernatant were progressively reduced when hStau-2 specific siRNA was used in a dose dependent manner. The experiments were done more than 3 times and error bars represents ± SD. *p value ≤ 0.05 were taken as significant.

With the observation that hStau-2 expression is promoted during HIV-1 infection in human astrocyte and T-lymphocyte cell lines, we studied the influence of hStau-2 overexpression on HIV-1 viral titers. hStau-2 was transiently expressed in HEK293T cells 24 hours prior to transfection with pro-viral DNA pNL4-3. HEK293T cells were transfected with increasing concentration of hStau-2-59 construct and protein expression was checked by Western blot using anti-hStau-2 antibody (Figure 4A, inset). The viral titers were measured by p24 ELISA after 48 hours of pNL4-3 transfection. It was observed that the overexpression of hStau-2 significantly increased the viral titers in a dose dependent manner (Figure 4A). As compared to the control experiment, p24 equivalent of viral titers increased by 56.6 ± 10.28% and 98 ± 3.85% when hStau-2 was overexpressed using 0.5 μg and 1 μg of hStau-2-59 construct respectively (Figure 4A). To check if the over-expression of hStau-2 is influencing the viral transcription and increasing the viral output, the total levels of full length viral transcripts were compared between hStau-2 over-expressed and control experiments. No significant changes at the level of full length transcripts were observed with respect to the control in our experiments (Figure 4B). Hence, the impact of overexpression of hStau-2 on viral transcription was ruled out.

The effect of hStau-2 on viral titers was further confirmed by siRNA mediated knockdown of hStau-2. hStau-2 specific siRNA and scrambled siRNA were used for the study. hStau-2 specific siRNA effectively knocked down hStau-2 transcript levels as quantified through semi-quantitative RT-PCR (Figure 4C, inset). A dose dependent reduction in hStau-2 transcript levels was observed when 20 pmoles and 50 pmoles of hStau-2 specific siRNA were used as compared to scrambled siRNA (Figure 4C, inset). To check the consequences of hStau-2 knockdown on HIV progeny synthesis, HEK293T cells were transfected with pro-viral DNA pNL4-3 after 24 hours of silencing. Cells were harvested after 48 hours of pNL4-3 transfection and HIV-1 p24 levels from the culture supernatants were measured by p24 ELISA (Figure 4C). The knockdown of hStau-2 resulted in a significant decrease in HIV-1 titers showing 30.75 ± 8.9% and 57.32 ± 11.82% reduction in p24 equivalent of viral titers in a dose dependent manner (Figure 4C). Together, these data confirmed that hStau-2 behaved as a positive regulator of HIV-1 production.

### hStau-2 increased RRE containing viral mRNA export activity of Rev

**Figure 5:**
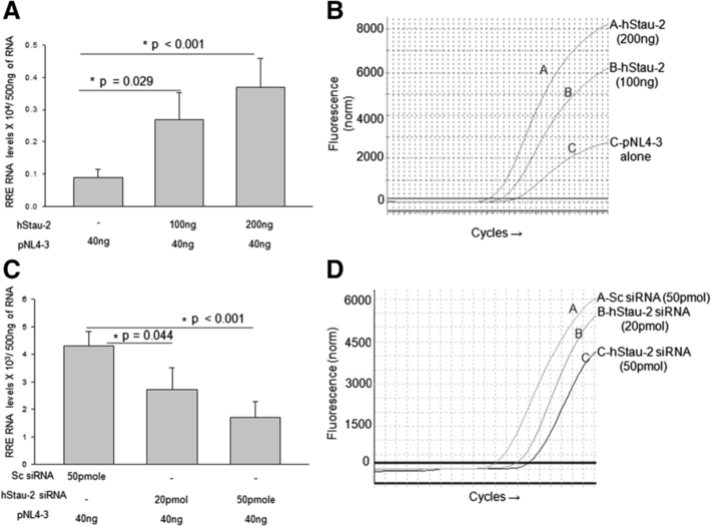
**Effect of hStau-2 over-expression and knockdown on Rev export activity. A)** Over-expression of hStau-2 increased RRE-RNA levels in the cytoplasmic fraction of HEK293T cells: hStau-2 over-expressed HEK293T cells or control cells were transfected with pNL4-3 pro-viral DNA. After 24 hrs of pNL4-3 transfection, total RNA was isolated from the cytoplasmic fractions and cDNA was prepared from 500 ng of RNA. Viral RRE was measured in the cytoplasmic fraction by absolute qRT-PCR. **B)** A leftward shift in CT curves for RRE-containing RNA upon hStau-2 overexpression. **C)** hStau-2 knockdown reduced RRE-RNA levels in the cytoplasmic fraction of HEK293T cells: hStau-2 siRNA or scrambled siRNA were transfected into HEK293T cells followed by pNL4-3 pro-viral DNA transfection. After 24 hrs of pNL4-3 transfection, total RNA was isolated from the cytoplasmic fractions and cDNA was prepared from 500 ng of RNA. Viral RRE was measured in the cytoplasmic fraction by absolute qRT-PCR. **D)** A rightward shift in CT curves for RRE-containing RNA upon hStau-2 knockdown. The experiments were done more than 3 times and the error bar represents ± SD. *p value ≤ 0.05 was taken as significant.

With the experiments confirming the promotion of HIV-1 production by hStau-2 in addition to its direct interaction with Rev, we studied whether hStau-2 can enhance the RNA export activity of Rev. hStau-2 was overexpressed in HEK293T cells followed by transfection with pro-viral DNA pNL4-3. After 24 hours of transfection, the cells were harvested and cytoplasmic fractions were separated. Total RNA from the cytoplasmic fractions were extracted. The purity of cytoplasmic fraction was confirmed by the absence of pre-GAPDH mRNA in RT-PCR (Additional file 3: Figure S3). The levels of RRE containing viral RNA in the cytoplasm were then quantified by real time PCR using RRE specific primers. To quantify the RRE levels, a standard plot was generated based on the copy numbers of the amplified RRE region from the known concentration of RRE-GEM construct used as a template (Additional file 4: Figure S4A-B). Compared to the control experiments, hStau-2 overexpression promoted the export activity of Rev in a dose dependent manner as evident by 3 and 4 fold increase in the levels of RRE containing viral RNA in the cytoplasmic fraction of HEK293T cells (Figure 5A). The CT values showed a significant shift towards the left with increasing amounts of hStau-2 expressing vector, indicating high copy number of RRE-RNA in the respective cytoplasmic fractions (Figure 5B).

To reconfirm that RRE-RNA export activity of Rev was indeed promoted by hStau-2, endogenous hStau-2 was knocked down using siRNA and the above experiment was repeated. HEK293T cells treated with either scrambled siRNA or hStau-2 specific siRNA (in two increasing amounts) were transfected with equal amount of pNL4-3 pro-viral DNA. 24 hours post pNL4-3 transfection, the RRE-containing viral RNA was quantified from the cytoplasmic fractions of these cells by qRT-PCR and the CT values were compared (Figure 5C-D). As clear from the plot in Figure 5C, RRE containing viral RNA in the cytoplasm of the cells were 1.5 and 2.5 fold less in conditions where hStau-2 was knocked down using increasing doses of siRNA (Figure 5C). As expected, a significant right shift in CT curves for RRE-containing RNA in hStau-2 knocked down cells were observed when compared to the scrambled siRNA treated cells (Figure 5D). Collectively, these experiments established that the host protein hStau-2, which was upregulated during HIV-1 infection, promoted RRE containing RNA export activity of Rev and increased HIV-1 titers.

### hStau-2 mutant defective in Rev interaction failed to promote RNA export activity of Rev and viral production

**Figure 6:**
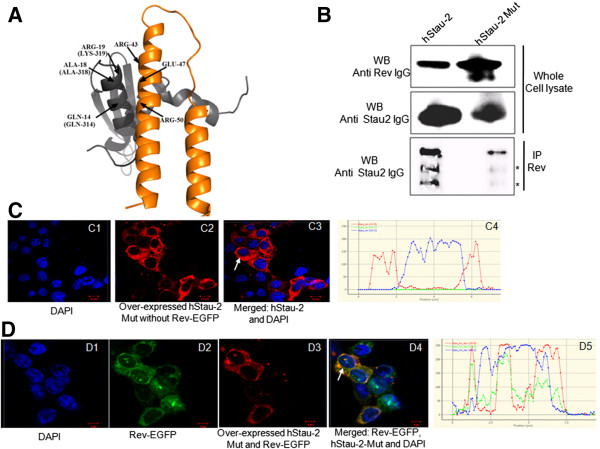
**Rev-hStau-2 interactions are essential for hStau-2 dependent promotion of Rev activity. A)** The docked complex of Staufen homolog from mouse (1UHZ, grey ribbon) and Rev protein (2X7L, orange ribbon) showing interacting residues: The corresponding residues of hStau-2 protein are indicated in brackets. **B)** Co-immunoprecipitation of hStau-2 or hStau-2Mut with Rev using anti-Rev antibody: HEK293T cells were transfected with Rev-EGFP vector and co-transfected with hStau2-59 or hStau-2Mut-CMV vector, cell lysates were prepared after 48 hours followed by IP and WB. Whole cell lysates were checked for Rev expression by anti-Rev antibody and for hStau-2 expression by anti-hStau-2 antibody. * indicates non-specific bands or degraded protein. **C)** Localization of hStau-2Mut. hStau-2Mut localizes in the cytoplasm: HEK293T cells were transfected with hStau-2Mut-CMV vector and stained with goat anti-Staufen-2, Rabbit anti-goat Alexa Fluor 568 antibody and nucleus was stained by DAPI. Distribution plot is shown at the end of the panel. **D)** Co-localization of Rev and hStau-2Mut: HEK293T cells were transfected with Rev-EGFP and hStau-2Mut-CMV vector. Localization of GFP-tagged Rev was determined by green fluorescence, hStau-2Mut was determined by goat anti-Staufen-2, Rabbit anti-goat Alexa Fluor 568 antibody and nucleus was stained by DAPI. Merged images show co-localization of hStau-2Mut and Rev. Distribution plot is shown at the end of the panel. The precise cells whose graphical analyses have been shown are indicated by the white arrows.

The experiments so far indicated that the presence of hStau-2 positively regulated Rev RNA export activity and the viral production. To confirm that the physical interaction between hStau-2 and Rev is instrumental in promoting Rev activity, a hStau-2 mutant (referred as hStau-2Mut) defective in Rev binding was generated. For this, HIV-1 Rev was docked onto available Staufen-2 homolog to predict the critical residues of hStau-2 that possibly interacted with Rev. The best ranked complexes from three different docking studies (Additional file 5: Figure S5 A-B) suggested that hStau-2 residues spanning 314–320 are playing an important role in the interaction with Rev (Figure 6A). Based on these predictions, hStau-2Mut with mutations Q314R, A318F and K319E was generated. This mutant was used in the co-immunoprecipitation assays to check for its interaction with Rev. Rev-GFP was co-overexpressed with either the wt hStau-2 or hStau-2Mut. The cell lysates were confirmed for the overexpression of Rev, wt hStau-2 and hStau-2Mut by Western blots (Figure 6B, 1^st^ and 2^nd^ panel). Immunoprecipitations were performed using mouse anti-Rev antibody. hStau-2 was detected by Western blot in the immunoprecipitated samples using goat anti-hStau-2 antibody (Figure 6B, 3^rd^ panel). It was observed that mutations, Q314R, A318F and K319E, did not obliterate the Rev-hStau-2 interactions completely, though made it defective for Rev binding (Figure 6B).

In order to check if hStau-2Mut defective for Rev binding had different sub-cellular distribution than the wt hStau-2, we observed its localization in the presence or in the absence of Rev by confocal microscopy (Figure 6C-6D). Pattern of distribution of hStau-2Mut in the sub-cellular regions of the nucleus and the cytoplasm was similar to that of the wild type hStau-2. Similar to the wild type hStau-2, hStau-2Mut localized primarily to the cytoplasm in the absence of Rev while a fraction of it localized to the nucleoli in the presence of Rev (Figure 6C-6D). These studies clearly indicated that though hStau-2Mut was defective in binding to Rev, other properties like the sub-cellular localization was not affected.

**Figure 7:**
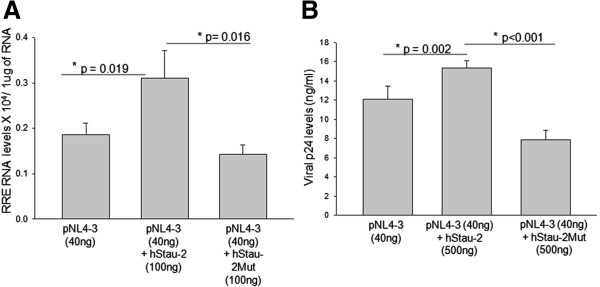
**Rev-hStau-2 interactions are essential for hStau-2 dependent promotion of Rev activity.** Viral RRE-RNA levels were measured in the cytoplasmic fraction by absolute qRT-PCR for either hStau-2 or hStau-2Mut over-expressing cells. **A)** hStau-2Mut did not promote RRE-RNA export activity of Rev: Viral RRE-RNA levels were measured in the cytoplasmic fraction by absolute qRT-PCR for hStau-2, hStau-2Mut over-expressing cells and the control cells. Concentrations of the plasmid hStau-2 or hStau-2-Mut was 0.1ug and pNL4-3 was 40 ng. **B)** Viral p24 levels were scored by p24 ELISA. Concentration of the plasmid hStau-2 or hStau-2-Mut was 0.5ug and pNL4-3 was 40 ng. hStau-2Mut did not increase HIV-1 production.

We then scored the influence of hStau-2Mut on the RNA export activity of Rev. The levels of RRE-containing viral RNA in the cytoplasm of HEK293T cells transfected with pNL4-3, overexpressing either the wild-type (hStau-2) or the mutant (hStau-2Mut) hStau-2 were studied using qRT-PCR (Figure 7A). It was evident that the cytoplasm of HEK293T cells over-expressing hStau-2 had higher levels of RRE-containing viral RNA compared to the control experiment (pNL4-3) denoting Rev-supporting function of hStau-2, while the levels of RRE-containing viral RNA in the cytoplasm of HEK293T cells that over-expressed hStau-2Mut remained similar to that of the control experiment (Figure 7A). The CT curve for RRE-containing RNA for hStau-2 showed a significant left shift with reference to the CT curve for the control experiment (Additional file 6: Figure S6 curves A and C), while no such shift was observed for hStau-2Mut (Additional file 6: Figure S6 curve B). From these results, it was inferred that hStau-2 dependent promotion of Rev activity was not exhibited by hStau-2Mut that was defective in interacting with Rev. Further, when p24 equivalent of HIV-1 titers was measured in the culture supernatants from similar experiments, it was observed that hStau-2 over-expressing cells produced more viruses than the control experiment, while cells expressing hStau-2Mut produced less virus particles, even lesser than that produced in the control experiment (Figure 7B). These experiments confirmed that the protein-protein interaction between Rev and hStau-2 was important for the modulation of Rev-export activity and viral titers.

## Discussion

This study describes a new host factor, Staufen-2, that assists in HIV-1 pathogenesis by supporting the activity of a critical viral regulatory protein, Rev, thereby increasing the viral production in an infected cell. The observations were supported by over-expression and siRNA mediated knockdown of hStau-2, where it was evident that hStau-2 increased the RRE-RNA export activity of Rev and the viral titers (Figures 4 and 5). Abrogating the direct interaction between Rev and hStau-2 by generating hStau-2 mutant defective for Rev binding, abolished hStau-2 dependent promotion of Rev activity and hence final viral output. This positive regulator of HIV-1 was also observed to be upregulated during HIV-1 infection of human T-lymphocyte and astrocyte cell lines (Figure 3A-B).

Cellular protein, Staufen belongs to dsRNA binding protein family, first identified in Drosophila [37]. Mammals possess two Staufen homologs, Staufen 1 and Staufen 2, each with multiple isoforms and splice variants [38]. Both the Staufen homologs are reported to form RNA-protein complexes and participate in transport, stability and decay of mRNA in human neurons or other polarized cells thereby regulating the spatial and the temporal gene expressions [30,39]. Recently, it was deciphered that Staufen 1, despite being a host protein, is selectively packaged in HIV-1 infectious particle through its interaction with HIV-1 Gag [34,35], while there were no previous reports of hStau-2 being involved in any phase of the HIV-1 life cycle. It was intriguing to identify hStau-2 in the pull-down samples of Rev as we noticed that Rev, a protein from the pathogen and hStau-2, a protein from the host, had overlapping biochemical and functional properties. Both the proteins bind RNA, shuttle between the nucleus and the cytoplasm and mediate mRNA transportation across the nuclear membranes. Earlier reports by independent studies showed that CRM1/Exportin-1 can interact separately with both Rev and hStau-2 and promote their RNA export activity [32,40]. With our studies showing that Rev and hStau-2 physically interacted with each other (Figure 1A and B) and that this interaction promoted the Rev activity (Figure 5), it can be speculated that together with CRM1 and hStau-2, Rev may form an elaborate export complex for the successful transportation or regulated release of large viral RNA across the nuclear membrane. It should be noted that the composition of such multiprotein-RNA complexes may vary across cell types and would be interesting to explore how the composition of such complexes impact the viral RNA transport by Rev.

A number of RNA binding host proteins like RNA helicase A (RHA), human Rev-interacting protein (hRIP) etc., interact with Rev and influence its activity [15,41,42]. These interactions are either direct or mediated by other proteins or RNA. In case of hStau-2, it could be established that the interaction with Rev is independent of RNA (Figure 1C-1D). Though this strongly suggested a direct protein-protein interaction, we cannot rule out the possibility of cellular or viral RNA existing between the two proteins under physiological conditions. Yet another interesting fact to further decipher would be to identify the respective RNA partners of hStau-2 and Rev under varied physiological conditions in different HIV permissive host cells.

In agreement with the positive regulation of hStau-2 on Rev activity, over-expression of hStau-2 increased viral titers. The essentiality of the direct protein-protein interaction between Rev-hStau-2 was established through hStau-2Mut defective in Rev binding that did not support the hStau-2 dependent promotion of Rev activity and increase in the viral titers (Figure 7A-B). In fact, the cells overexpressing hStau-2Mut produced lesser virus than pNL4-3 alone transfected control cells (Figure 7B). These observations are suggestive of multiple roles of hStau-2 in the life cycle of HIV-1 apart from increasing Rev mediated viral RNA export activity. These studies also demonstrated that disrupting the protein-protein interaction between Rev and hStau-2 can prevent hStau-2 from helping viral production. This information holds high significance as the Rev-Staufen2 docking studies (Figure 6A and Additional file 5: Figure S5) can be further exploited to design peptide based retroviral inhibitors where disrupting Rev-hStau-2 interactions would limit viral propagation.

Staufen containing RNA granules are a part of high molecular mass (HMM) complexes of cytoplasmic nucleoproteins. Low Molecular Mass (LMM) A3G when gets converted into HMM loses its antiretroviral property and cannot limit HIV-1 propagation in infected cells [43,44]. In our observations, hStau-2 expression was upregulated upon HIV-1 infection of CD4 + T lymphocyte cell line SUP-T1. It can be inferred that T-cells, when activated by HIV-1 infection expressed more hStau-2 that further helped making more Staufen containing HMM complexes that prevented endogenous LMM-A3G from inhibiting HIV propagation. This can be one more approach to explain the high viral production by the infected cells over-expressing hStau-2 apart from the pro-Rev function of hStau-2. HIV-1 infection upregulated hStau-2 expressions in astrocytes as well (Figure 3B). It would be interesting to further study the impact of increased levels of hStau-2 on neuronal cells. Distorted hStau-2 levels in these cells upon HIV infection may alter mRNA transport and hence protein expressions that can explain conditions like HIV associated neuro-dementia.

This study has established the involvement of yet another member of the Staufen family of protein in HIV pathogenesis, where hStau-2 regulated HIV-1 titers through direct interaction with one of the key viral proteins. It is evident from our study that one possible mechanism by which hStau-2 promoted viral production is by aiding HIV-1 protein Rev in transporting viral RNA for the synthesis of late viral structural proteins. Further investigations to elucidate the mode of action of hStau-2 in context of HIV pathogenesis will add substantial information for designing new anti-retroviral strategies.

## Conclusion

Conclusively, we have identified host protein hStau-2 as a positive effector of HIV-1 Rev that promoted its RNA export activity, thereby facilitating viral production, the expression of which was upregulated upon HIV-1 infection.

## Methods

### Cell lines, viral stock production and HIV-1 infection

The cell lines used in the study: HEK293T (human embryonic kidney cell line) obtained from Dr. Reddy’s Institute of Life Sciences, Hyderabad and Astrocyte 1321 N1 human astrocytoma (gift from Dr. Kondapi) were maintained in DMEM media (Invitrogen, USA). SUP-T1 (human T cell lymphoblastoma) obtained from Dr. S. Jameel, ICGEB Delhi was maintained in RPMI-1640 media (Invitrogen, USA). Additionally, the media were supplemented with 10% fetal bovine serum (Gibco, USA), 100 U of penicillin/ml and 100 μg of streptomycin/ml (*Himedia Laboratories*, India). All the cell lines were grown in 37°C with 5% CO_2_.

Infectious HIV-1 particles were produced by transfection of proviral DNA pNL4-3 into HEK293T cells by calcium phosphate method [45]. Culture supernatants were collected at an interval of 24 hours post transfection, filtered through 0.45 μM syringe filter (Millipore, USA), precipitated using Polyethylene glycol [45] and quantified by HIV-1 p24 ELISA kit (Advanced BioScience Laboratories Inc, USA) according to the manufacturer’s protocol. The virus thus produced is HIV type 1 (HIV-1) referred as NL4-3. Cells were infected with 50 ng/ml of p24 equivalent of NL4-3 virus in the presence of 8 μg/ml of polybrene (Sigma-Aldrich, USA). After 4 hours of infection, cells were washed with phosphate buffer saline (PBS) and kept for 48 hours in complete media. The protocol used and standards maintained are approved by Institutional Biosafety Committee, University of Hyderabad.

### Plasmids and constructs

#### His-Rev-setB

Full length cDNA of HIV-1 Rev was amplified from CMVsrev (gift from Prof. B. K. Felber, Center for Cancer Research, USA) using primers Rev-His-FP and Rev-His-RP (Additional file 7: Table S1) and cloned into BglII and NcoI sites of pRSET-B *E.coli* T7 Expression Vector (Invitrogen, USA) to generate hexa-histidine tag at the N-terminal.

#### Rev-EGFP-C3

The Rev gene was amplified from His-Rev-setB construct using primers Rev-GFP-FP and Rev-GFP-RP (Additional file 7: Table S1) and cloned into BglII and SalI site of pEGFP-C3.

#### hStau-2Mut-CMV

hStau-2-59 (gift from Prof. Luc Desgroseillers, Université de Montréal, Canada) was used as a template to create Human Staufen 2 mutant. The mutant region containing Q314R, A318F and K319E mutations were generated by overlap PCR using Stau-2Mut FP and Stau-2Mut RP (Additional file 7: Table S1) and cloned into SalI and XbaI site of pCMV-Sport 1 (Invitrogen, USA).

#### RRE-GEM

Using pNL4-3 as a template, RRE sequence was cloned into EcoRI and HindIII sites of pGEM-3Zf + (Promega, USA) vector. The primer sequences used were RRE-FP and RRE-RP (Additional file 7: Table S1).

All the constructs were confirmed by sequencing (Eurofins, India).

Other plasmids used were hStau-2-59 (gift from Prof. Luc Desgroseillers, Université de Montréal, Canada) for expression of wild type hStau-2, pro-viral DNA construct pNL4.3 (gift from Prof. A. K. Kondapi, University of Hyderabad, India), pEGFP-C3 (Clonetech, USA).

### Expression and purification of the recombinant Histidine tagged Rev protein

His-Rev-setB construct was transformed into BL-21 DE3 codon plus (RIL) cells (Agilent technologies, USA) for expressing recombinant Rev protein with N terminal 6X histidine tag by induction with 1 mM of Isopropyl β-D-1-thiogalactopyranoside (IPTG) (Fermentas, Germany) for 6 hours at 37°C. Rev was purified under native conditions using Talon-affinity resin (Clontech, USA) according to the manufacturer’s protocol and eluted with 150 mM imidazole. After purification, Rev protein was dialyzed in modified Rev-buffer [46] with the composition of 50 mM sodium phosphate buffer, pH 7.0, 150 mM NaCI, 10 mM K_2_S0_4_, and 1 mM DTT. The protein was concentrated using centricon with 3 kDa cutoff (Millipore, USA). Protein concentration was measured by 1X Bradford Dye Reagent (Bio-Rad, USA) according to the manufacturer’s instructions. The purified protein was checked on 15% SDS PAGE (Additional file 1: Figure S1A) and confirmed by Western blot using 1:2000 dilution of mouse anti-Rev antibody (SantaCruz Biotechnology Inc., USA) and 1:2500 dilution of HRP conjugated goat anti-mouse IgG antibody (SantaCruz Biotechnology Inc., USA) followed by detection with Pierce ECL western blotting substrate (Thermo Scientific, USA) and visualized using VersaDoc gel imaging system (BioRad, USA).

### Affinity chromatography to identify Rev interacting host factors

Affinity column was prepared using recombinant His-Rev protein according to the previously described protocol [47] with some modifications. In brief, 100 μg of recombinant His-Rev was added to the 500 μl of Talon resin and incubated for 4 hours at 4°C. Wash with Rev buffer (50 mM sodium phosphate buffer, pH 7.0, 150 mM NaCI, 10 mM K_2_S0_4_, and 1 mM DTT) was given to remove the unbound Rev protein. SUP-T1 cells were lysed by lysis buffer (50 mM Tris–HCl pH 7.5, 150 mM NaCl, 2% Glycerol, 5 mM DTT, 0.1% NP-40, 1 mM PMSF and protease inhibitor cocktail), centrifuged to remove debris and supernatant from which 1 mg of total protein was added to the Rev bound Talon resin. As a control, SUP-T1 cell lysates were added to the column without bound recombinant His-Rev protein. After overnight incubation, columns were washed 5 times with wash buffer (Rev buffer + 50 mM imidazole). The samples were eluted using elution buffer (Rev buffer + 150 mM imidazole). 40 μl of eluted samples from both test and control columns were subjected to SDS PAGE followed by silver staining [48]. Bands unique to Rev affinity column pull-down were excised (Additional file 1: Figure S1B) and identified using MALDI-TOF (Bruker Daltonics, Bremen, Germany) at proteomics facility of the University of Hyderabad, India. Protein bands were identified using database searches (PMF and MS/MS) using MASCOT program by Biotools software (Bruker Daltonics) (Additional file 1: Figure S1C and Additional file 2: Figure S2).

### Immunoprecipitations

Transfected HEK293T cells were washed with PBS and lysed in IP-Buffer [20 mM Tris HCl pH 8.0, 137 mM NaCl, 10% glycerol, 1% Nonidet P-40 (NP-40)]. Cells were sonicated and centrifuged at 12000 rpm at 4°C to remove cellular debris. For RNA digestion, cell lysates were treated either with RNase A or with RNase III as per the manufacturer’s protocol. Agarose A/G beads (SantaCruz Biotechnology Inc., USA) were washed with IP buffer and incubated with cell lysates for 2 hours at 4°C to remove proteins that non-specifically bind to the beads (pre-clearing step). After pre-clearing, the beads were centrifuged at 4000 rpm and the supernatant was added to the new column containing A/G beads already conjugated with mouse anti-Rev antibody or anti-GFP antibody for 4 hours at 4°C on a rocker. After 4 hours of incubation, samples were washed 3 times with IP buffer. The samples were processed for Western blot.

### Western blot

Samples were fractionated on 10% SDS–PAGE and transferred to methanol treated PVDF membrane (GE-Amersham, USA) in a Western blot transfer apparatus. After the membranes were blocked with 5% non fat milk for 1 hour at RT, the primary antibody was added to the membrane and incubated overnight at 4°C on a rocker. The antibody dilution used for mouse anti-Rev antibody was 1:1000 (SantaCruz Biotechnology Inc., USA), for mouse anti-GFP antibody was 1:1000 (SantaCruz Biotechnology Inc., USA) and for goat anti-Stau-2 antibody was 1:500 (SantaCruz Biotechnology Inc., USA). After 3 washes with PBS-T (PBS + 0.05% Tween 20), the membranes were incubated with the respective HRP conjugated antibodies namely, goat anti mouse IgG with 1:2000 dilution (SantaCruz Biotechnology Inc., USA) and donkey anti-goat IgG with 1:1000 dilution (SantaCruz Biotechnology Inc., USA) for respective detection of Rev, GFP and hStau-2 at RT for 1 hour. Following 3 washes with PBS-T, ECL substrate (Super Signal West Femto Chemiluminescent Substrate Pierce) was added to the membranes and was scanned using Versadoc system (Biorad). The Western blot images were quantified by Image-J software (NIH).

### Immuno-fluorescence microscopy

For fluorescence studies, cells were grown on coverslips one day before transfection. Cells were transfected with respective plasmids using lipofectamine LTX plus reagent (Invitrogen, USA). After 48 hours of transfection, cells were washed twice with PBS and fixed in 3% paraformaldehyde. Fixed cells were washed 3 times with PBS to remove excess of paraformaldehyde and permeabilized by adding ice-cold methanol for 5 min at −20°C. Cells were washed thoroughly and mounted with DAPI. Rev-GFP exhibited green fluorescence. hStau-2 was detected using polyclonal anti-hStau-2 antibody and corresponding secondary fluorescent-tagged, Alexa Fluor® 568 rabbit anti-goat IgG antibody (red). The nuclei of the cells were stained with DAPI (blue). Cells were seen under Leica confocal TPS2 microscope at 20X magnification and analyzed using Leica software. For localization in the cytoplasm or the nucleus, several fields were scanned and Huygens Essential software was used to plot graph representing fluorescence intensities of blue (for nucleus), green (for Rev) and red (for Staufen-2) channels across the cells using twin slicer tool [24].

### Quantitative Real time PCR

RNA was isolated using Trizol reagent (Invitrogen, USA) according to the manufacturer’s protocol. cDNA was prepared from RNA by using Superscript III (Invitrogen, USA) and oligo dT (Fermentas, Germany ) at 50°C for 1 hour followed by inactivation at 70°C for 15 min. hStau-2 and β-actin genes were amplified by SYBR Premix ExTaq (Takara Bio Inc, Japan) in Eppendorf Mastercycler Realplex^2^ (Eppendorf, Germany). The fold difference was calculated using the formula 2^-(ΔΔCT)^. The primers used were Stau-RT-FP, Stau-RT-RP, Actin-RT-FP and Actin-RT-RP mentioned in Additional file 7: Table S1.

### Transfection and silencing

HEK293T cells were seeded one day before transfection at 70% confluency. Cells were washed with PBS and transfected with plasmids using Lipofectamine-LTX plus reagent (Invitrogen, USA). After 48 hours of transfection, cells were harvested.

To silence h*stau-2* gene, a 21 bp duplex siRNA was synthesized for a selected region of hStau-2. As a control, scrambled siRNA was used. hStau-2 specific siRNA (20 and 50 picomoles) or scrambled siRNA (50 picomoles) were transfected using Lipofectamine RNAiMAX reagent (Invitrogen, USA). The synthesized siRNA were tested for knockdown efficiency in preliminary experiments by semi-quantitative RT-PCR using Stau-RT-FP and Stau-RT-RP primers for 25 cycles (Additional file 7: Table S1). β-actin was used as a control for the experiment. The reaction without reverse transcriptase was used as a negative control to confirm that RNA preparation is free of DNA contamination.

After 24 hours of siRNA treatment, cells were transfected with pNL4-3 plasmid and kept for additional 48 hours.

### Quantification of total viral transcripts

For quantification of total viral RNA transcripts, equal amount of cDNA was used to amplify 9 kb product by qRT-PCR using primer set TAR-FP and 9 kb-RP (Additional file 7: Table S1) as described earlier [34]. The fold difference was calculated using the formula 2^-(ΔΔCT)^ and normalized with β-actin levels.

### Quantification of RRE-RNA export activity of Rev

HEK293T cells were transfected with hStau2-59 with lipofectamine LTX plus reagent (Invitrogen, USA) for overexpression. After 24 hours of rest, cells were transfected with equal amount of pNL4-3. After 48 hours of hStau-2 transfection, cells were harvested and cytoplasmic RNA was isolated using Paris kit (Ambion, USA). RNase free DNase I treatment was given to remove DNA contamination. 2 μg of RNA from each group were reverse transcribed using SuperScript III Reverse Transcriptase (Invitrogen, USA), random hexamer at 50°C for 1 hour followed by inactivation at 70°C for 15 min. For absolute real time PCR, standard was made using serial dilution of RRE-GEM construct containing known copy numbers (Additional file 4: Figure S4). The FAM labeled RRE taqman probe was commercially designed and synthesized (Invitrogen, USA). 500 ng of cytoplasmic RNA was reverse transcribed and then PCR amplified using probe, taqman universal mix II with UNG (Invitrogen, USA) and checked for the number of copies of RRE representative of unspliced RNA. UNG glycosylase was added to remove any carryover of PCR. Background reading was checked by analyzing negative RT control. Nuclear contamination in cytoplasmic fraction was checked by PCR amplification by pre-GAPDH primers (Additional file 3: Figure S3).

### HIV-1 Rev-hStau-2 protein-protein docking

The structure of the Rev protein is available in PDB (2X7L), and chain M of this protein was used to perform the docking study with Staufen homologs. As the structure of hStau-2 is not available, the sequence of hStau-2 was used in a BLASTP search [49] against PDB for finding suitable homologs. The homology search revealed that the N-terminal of the hStau-2 protein (Residues 6 to 70) had 39% sequence similarity with Dsrm domain of spermatid perinuclear RNA-binding protein of human (2DMY), amino acid residues 201–275 had 57% identity with the Staufen protein from Drosophila (1EKZ) and amino acid residues 308–383 had 96% identity with the dsRNA binding domain in Staufen homolog (PDB id: 1UHZ) from mouse. Three different docking studies were performed between the known Rev structure and each of the homologs obtained for hStau-2. PatchDock [50,51] server with refinement tool FireDock [52,53] were used for molecular docking. PatchDock provided results which were ranked according to geometric shape complementarity score after molecular shape representation and surface patch matching. These results were used for further refinement and re-scoring of 1000 top scoring complexes using FireDock. The results from Firedock ranked the complexes according to the global energy score. The best docking model with the Staufen homolog of mouse (1UHZ) and HIV-1 Rev (2X7L) is shown in Additional file 5: Figure S5B and Figure 6A. The global energy function of this complex was calculated by FireDock server to be −7.05 of relative units (this value is considered to be related to free binding energy and higher negative value means higher free binding energy and thus higher interaction probability). Using this model, the residues which are forming intermolecular interactions like hydrogen bonds, electrostatic and hydrophobic interactions among these two protein structures were identified. The interacting residues between Rev and hStau-2 for this model were also predicted by cons-PPISP server [54,55]. The best ranked docked complexes between Staufen homolog of mouse (1UHZ) and HIV-1 Rev protein (2X7L) suggested that the possible residues of hStau-2 that can bind HIV-1 Rev resided in the regions 232–235, 255–262, 314–320 and 370–375. Subsequent mutation studies were carried out to validate this prediction. One such hStau-2 mutant, carrying the mutations Q314R, A318F and K319E, was found defective in binding Rev.

### Statistical analyses

All the experiments were performed more than three times or as mentioned in figure legends. Average values of the experiments are plotted and error bars represent ± SD (standard deviation). Statistical analyses were done with Student’s *t*-test and the significance expressed as p value ≤0.05.

## Abbreviations

HIV: Human immunodeficiency virus, (hStau-2): human staufen-2; qRT-PCR: Quantitative Real Time PCR; AIDS: Acquired immune deficiency syndrome; RRE: Rev response element; RNPs: Ribonucleoproteins; EGFP: Enhanced green fluorescent protein; HEK: Human embryonic kidney cells; MALDI-TOF: Matrix-assisted laser desorption/ionization time of flight; ELISA: Enzyme-linked immunosorbent assay; IP: Immunoprecipitation; siRNA: Small interfering RNA; oligos: Oligonucleotides; HRP: Horseradish peroxidase; DAPI: 4′,6-diamidino-2-phenylindole; IPTG: Isopropyl β-D-1-thiogalactopyranoside; DTT: Dithiothreitol; UNG: Uracil N-Glycosylase; PDB: Protein Data Bank; PMSF: phenylmethanesulfonylfluoride.

## Competing interests

The authors declare that there is no competing interests.

## Authors’ contributions

AB and SB conceived and designed the study; AB, RB, KB and PG performed the experiments; AB, RB and SB analyzed the data and wrote the paper. All authors have read and approved the final manuscript.

## Supplementary Material

Additional file 1: Figure S1 Identification of human Staufen-2 (hStau-2) as Rev interacting factor through affinity column chromatography. **A)** Purification of recombinant Histidine tagged Rev protein: Recombinant Rev protein was purified from BL-21 DE3 codon plus (RIL) cells and checked on 15% SDS PAGE followed by Coomassie Staining. Rev was purified to homogeneity and showed a band ~18 kDa. 1 and 2 denotes two batches of eluted purified. protein. **B)** Purified protein was used as bait to pull down Rev interacting factors from SUP-T1 lysates: Representative gel showing fractionation of pull down samples from 3 experiments (T1, T2 and T3). Lanes C1, C2 and C3 represent control experiments where SUP-T1 cell lysates were incubated with Talon resin without Rev. M represent protein marker and rec-Rev represents the purified protein. Arrows indicate the bands excised for MALDI analyses from this gel. The experiment was repeated more than 10 times and protein bands were excised from several gels and given for MALDI analysis. **C)** Table showing partial list of proteins identified as Rev interacting host factors from affinity chromatography experiments which were repeatedly identified in several pull down assays. The list included Staufen-2. Click here for file

Additional file 2: Figure S2 Mascot search result and score histogram for Staufen-2. Click here for file

Additional file 3: Figure S3 Reverse Transcriptase-PCR to check the purity of the cytoplasmic fractions: Cytoplasmic fractions were separated and RNA was isolated using PARIS kit. cDNA was prepared from isolated RNA and amplified by β-actin and pre-GAPDH primers as mentioned in the **Table S1**. Absence of pre-GAPDH band in cytoplasmic fraction is indicative of the purity of cytoplasmic fraction which showed amplified β-actin band and not pre-GAPDH band. Click here for file

Additional file 4: Figure S4 RRE standard plots. Concentration of RRE template (construct RRE-GEM) was determined spectrophotometrically and the number of copies was determined for each dilution. **A)** FAM labeled Taqman probe was designed against RRE. Fluorescence intensities of RRE standards were plotted against their respective CT values. **B)** Standard curve of CT value *vs* amount. This standard curve was used for determining copy numbers of unknown samples. Click here for file

Additional file 5: Figure S5 Docking studies to predict hStau-2 amino acid residues that can possibly interact with Rev: **A)** The alignment of hStau-2 with the mouse homolog of Staufen: The residues which are mutated are highlighted in the alignment. **B)** The docked complex of Staufen homolog (1UHZ) and Rev protein (2X7L): The HIV-1 Rev protein is depicted in ribbon and hStau-2 protein in spacefill to show the probable binding sites. The interacting residues in Staufen are shown as sticks and are labeled. Click here for file

Additional file 6: Figure S6 CT value curve for RRE export by wt hStau-2 and hStau-2Mut overexpression: A leftward shift in the CT curve of viral RRE levels upon hStau-2 overexpression (curve A) though no such shift is observed in hStau-2Mut overexpression which remained similar to the control experiment (curve B and C). Click here for file

Additional file 7: Table S1 Primers used in the study. Click here for file
