# Supplementary figures and images for: Human protein Staufen-2 promotes HIV-1 proliferation by positively regulating RNA export activity of viral protein Rev
Source: Retrovirology. 2014 Feb 13;11:18. doi: 10.1186/1742-4690-11-18 (PMC4016256; doi:10.1186/1742-4690-11-18)

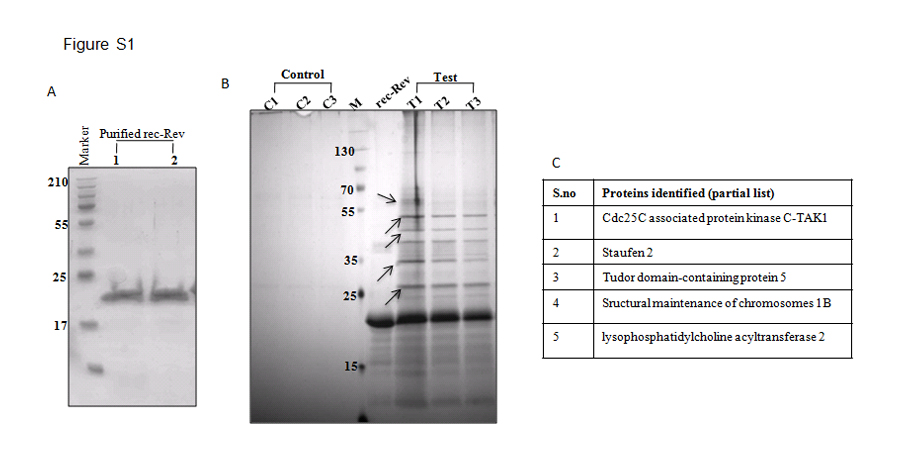

Supplement: Additional file 1: Figure S1 — Identification of human Staufen-2 (hStau-2) as Rev interacting factor through affinity column chromatography. A) Purification of recombinant Histidine tagged Rev protein: Recombinant Rev protein was purified from BL-21 DE3 codon plus (RIL) cells and checked on 15% SDS PAGE followed by Coomassie Staining. Rev was purified to homogeneity and showed a band ~18 kDa. 1 and 2 denotes two batches of eluted purified. protein. B) Purified protein was used as bait to pull down Rev interacting factors from SUP-T1 lysates: Representative gel showing fractionation of pull down samples from 3 experiments (T1, T2 and T3). Lanes C1, C2 and C3 represent control experiments where SUP-T1 cell lysates were incubated with Talon resin without Rev. M represent protein marker and rec-Rev represents the purified protein. Arrows indicate the bands excised for MALDI analyses from this gel. The experiment was repeated more than 10 times and protein bands were excised from several gels and given for MALDI analysis. C) Table showing partial list of proteins identified as Rev interacting host factors from affinity chromatography experiments which were repeatedly identified in several pull down assays. The list included Staufen-2. [file 1742-4690-11-18-S1.jpeg]

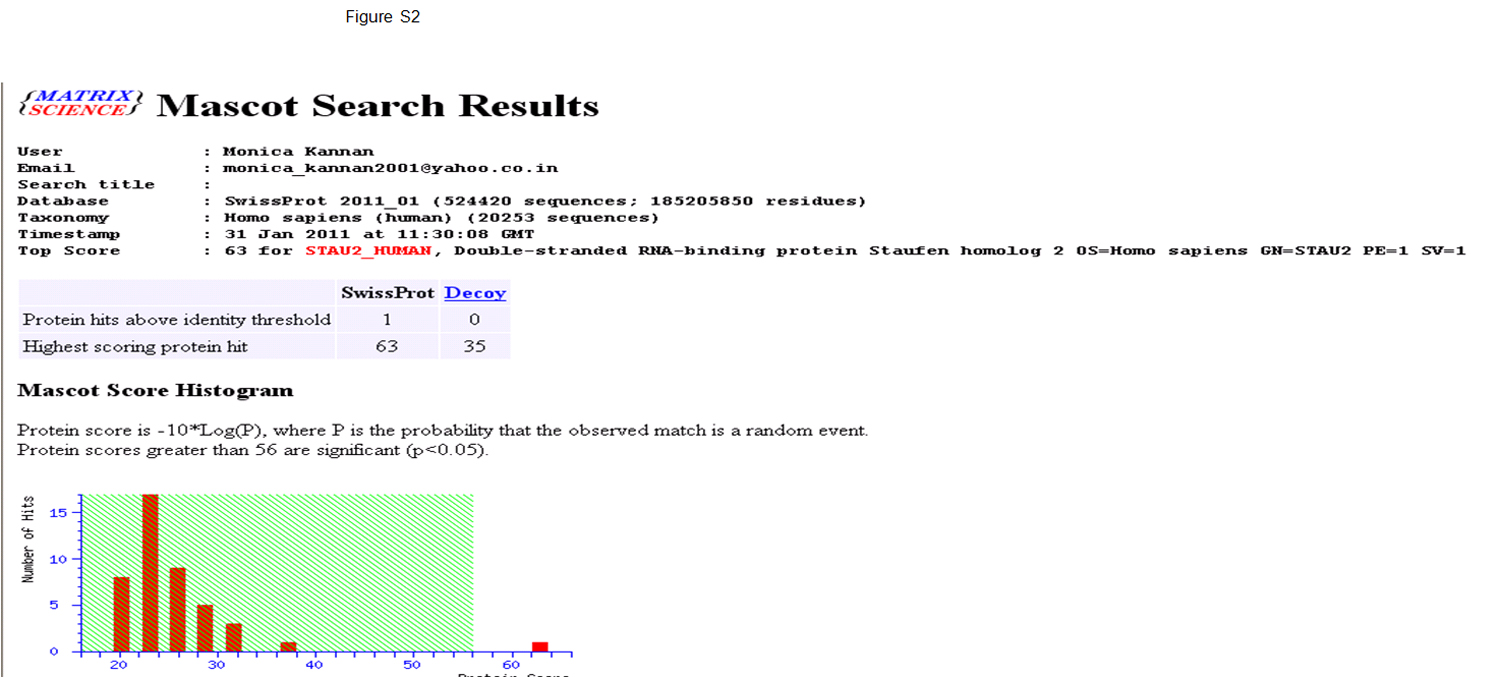

Supplement: Additional file 2: Figure S2 — Mascot search result and score histogram for Staufen-2. [file 1742-4690-11-18-S2.jpeg]

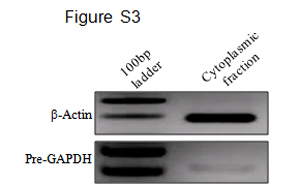

Supplement: Additional file 3: Figure S3 — Reverse Transcriptase-PCR to check the purity of the cytoplasmic fractions: Cytoplasmic fractions were separated and RNA was isolated using PARIS kit. cDNA was prepared from isolated RNA and amplified by β-actin and pre-GAPDH primers as mentioned in the Table S1. Absence of pre-GAPDH band in cytoplasmic fraction is indicative of the purity of cytoplasmic fraction which showed amplified β-actin band and not pre-GAPDH band. [file 1742-4690-11-18-S3.jpeg]

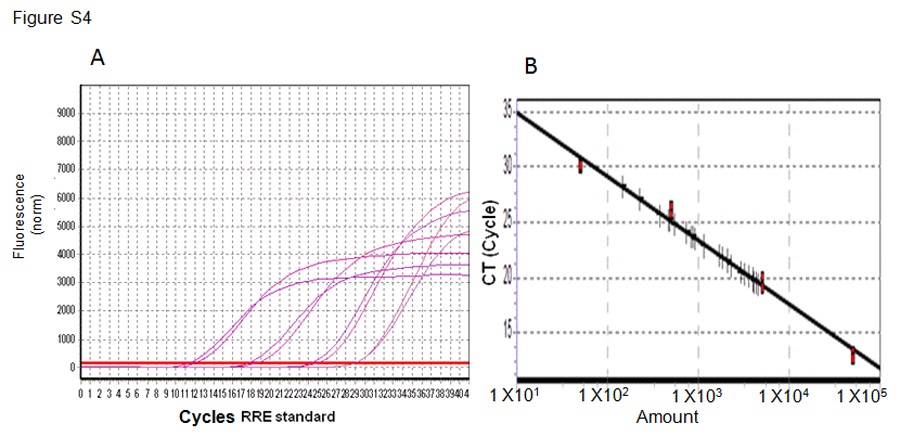

Supplement: Additional file 4: Figure S4 — RRE standard plots. Concentration of RRE template (construct RRE-GEM) was determined spectrophotometrically and the number of copies was determined for each dilution. A) FAM labeled Taqman probe was designed against RRE. Fluorescence intensities of RRE standards were plotted against their respective CT values. B) Standard curve of CT value vs amount. This standard curve was used for determining copy numbers of unknown samples. [file 1742-4690-11-18-S4.jpeg]

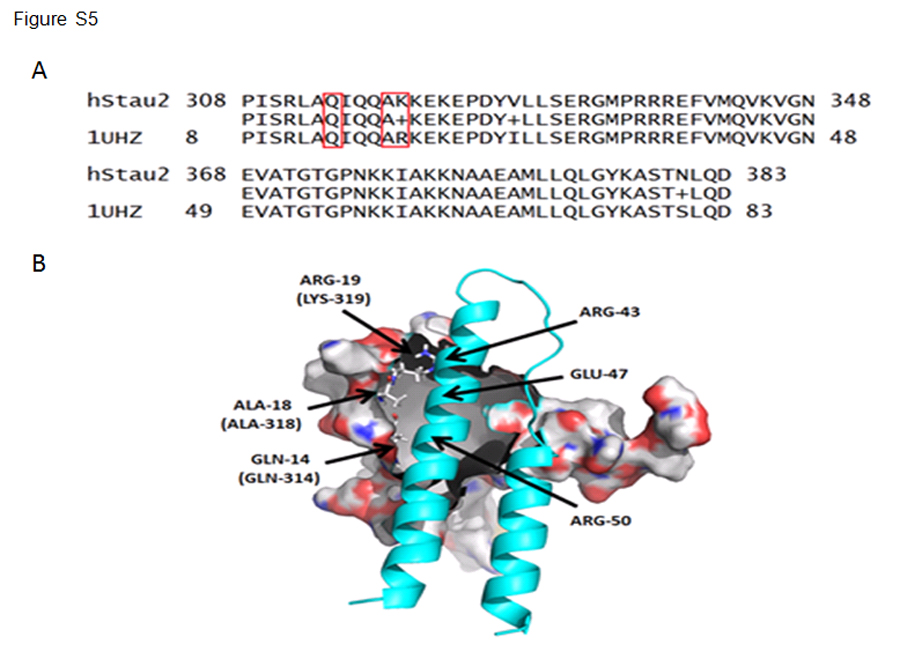

Supplement: Additional file 5: Figure S5 — Docking studies to predict hStau-2 amino acid residues that can possibly interact with Rev: A) The alignment of hStau-2 with the mouse homolog of Staufen: The residues which are mutated are highlighted in the alignment. B) The docked complex of Staufen homolog (1UHZ) and Rev protein (2X7L): The HIV-1 Rev protein is depicted in ribbon and hStau-2 protein in spacefill to show the probable binding sites. The interacting residues in Staufen are shown as sticks and are labeled. [file 1742-4690-11-18-S5.jpeg]

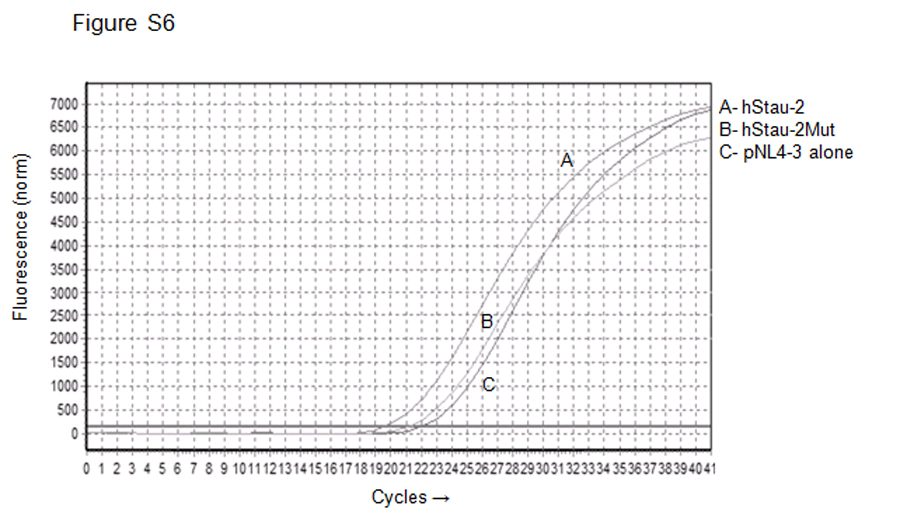

Supplement: Additional file 6: Figure S6 — CT value curve for RRE export by wt hStau-2 and hStau-2Mut overexpression: A leftward shift in the CT curve of viral RRE levels upon hStau-2 overexpression (curve A) though no such shift is observed in hStau-2Mut overexpression which remained similar to the control experiment (curve B and C). [file 1742-4690-11-18-S6.jpeg]
