# Supplementary material for: Human protein Staufen-2 promotes HIV-1 proliferation by positively regulating RNA export activity of viral protein Rev
Source: Retrovirology. 2014 Feb 13;11:18. doi: 10.1186/1742-4690-11-18 (PMC4016256; doi:10.1186/1742-4690-11-18)
Supplement: Additional file 7: Table S1 — Primers used in the study. [file 1742-4690-11-18-S7.docx]

**Table S1: Primers used in the study**

| Rev-His | Rev-His-FP | 5’ TCGAgaTctAtgGcaGgaAgaAgc 3’ |
| --- | --- | --- |
|  | Rev-His-RP | 5’ agtCCATGGtcaaattaattctctttagt 3’ |
| Rev-GFP | Rev-GFP-FP | 5’ ATCATAGATCTAATGGCAGGAAGAAGC 3’ |
|  | Rev-GFP-RP | 5’ CCCGTCGACAATTAATTCTCTTTAGTTC 3’ |
| RRE | RRE-FP | 5’ CATGAATTCGTGGGAATAGGAGCTTTGTTC 3’ |
|  | RRE-RP | 5’ CCCAAGCTTTTGATCCTTTAGGTATCTTTCCAC 3’. |
| hStau-2 Mut | Stau-2Mut FP | 5’ GCCGCCTGGCGCGAATTCAACAGTTCGAAAAGGAAA 3’ |
|  | Stau-2Mut RP | 5’ TTTCCTTTTCGAACTGTTGAATTCGCGCCAGGCGGC 3’ |
| hStau-2-RT | Stau-RT-FP | 5’ GCAGCCTTAAGTGCCTTGAAACAATT 3’ |
|  | Stau-RT-RP | 5’ AAACTCGAGCTAGACGGCCGAGTTTGATTTCTTGC 3’ |
| Actin-RT | Actin-RT-FP | 5’ AGCCTCGCCTTTGCCGA 3’ |
|  | Actin-RT-RP | 5’ CTGGTGCCTGGGGCG 3’ |
| Pre-GAPDH | P-GAPDH-FP | 5’ CCACCAACTGCTTAGCACC 3’ |
|  | P-GAPDH-RP | 5’ CTCCCCACCTTGAAAGGAAAT 3’ |
| 9kb viral RNA | TAR-FP | 5’ CTGAGCCTGGGAGCTCTCTGGC 3’ |
|  | 9kb-RP | 5’ TGCGAATCGTTCTAGCTCCCTGCTTGCCCATACTATATGTTT 3’ |
